# Microstructure and Hardness Properties of Additively Manufactured AISI 316L Welded by Tungsten Inert Gas and Laser Welding Techniques

**DOI:** 10.3390/ma17184489

**Published:** 2024-09-12

**Authors:** Mohamed Elsayed, Mahmoud Khedr, Antti Järvenpää, A. M. Gaafer, Atef Hamada

**Affiliations:** 1 Mechanical Engineering Department, Faculty of Engineering at Shoubra, Benha University, Cairo 11629, Egypt; 2 Future Manufacturing Technologies (FMT), Kerttu Saalasti Institute, University of Oulu, Pajatie 5, FI-85500 Nivala, Finland

**Keywords:** laser powder bed fusion, TIG welding, laser welding, 316L stainless steel, microstructure, hardness

## Abstract

In this study, 316L austenitic stainless-steel (ASS) plates fabricated using an additive manufacturing (AM) process were joined using tungsten inert gas (TIG) and laser welding techniques. The 316L ASS plates were manufactured using a laser powder bed fusion (LPBF) technique, with building orientations (BOs) of 0° and 90°, designated as BO-0 and BO-90, respectively. The study examined the relationship between indentation resistance and microstructure evolution within the fusion zone (FZ) of the welded joints considering the effects of different BOs. Microstructural analysis of the weldments was conducted using optical and laser confocal scanning microscopes, while hardness measurements were obtained using a micro-indentation hardness (H_IT_) technique via the Berkovich approach. The welded joints produced with the TIG technique exhibited FZs with a greater width than those created by laser welding. The microstructure of the FZs in TIG-welded joints was characterized by dendritic austenite and 1–4 wt.% δ-ferrite phases, while the corresponding microstructure in laser-welded joints consisted of a single austenite phase with cellular structures. Additionally, the grain size values of FZs produced using the laser welding technique were lower than those produced using the TIG technique. Therefore, TIG-welded joints showcased hardness values lower than those welded by laser welding. Furthermore, welded joints with the BO-90 orientation displayed the greatest cooling rates following welding processing, leading to FZs with hardness values greater than BO-0. For instance, the FZs of TIG-welded joints with BO-0 and BO-90 had H_IT_ values of 1.75 ± 0.22 and 2.1 ± 0.09 GPa, whereas the corresponding FZs produced by laser welding had values of 1.9 ± 0.16 and 2.35 ± 0.11 GPa, respectively. The results have practical implications for the design and production of high-performance welded components, providing insights that can be applied to improve the efficiency and quality of additive manufacturing and welding processes.

## 1. Introduction

The American Iron and Steel Institute (AISI) recognizes 316L austenitic stainless steel (ASS) as the predominant alloy within its category [1]. It contains up to 14 wt.% nickel (Ni), which stabilizes the austenite phase at lower temperatures and enhances corrosion resistance [2]. The low carbon content of 316L ASS improves weldability and reduces the risk of carbide precipitation, thereby maintaining corrosion resistance in welded structures [3]. Consequentially, it finds extensive application in aerospace and aircraft [4]. In recent years, the pursuit of lightweight of aerospace and aircraft components has emerged as a significant trend alongside the rapid growth of the aviation industry and additive manufacturing (AM) [5]. AM encompasses seven categorized techniques, among which laser powder bed fusion (LPBF) stands out as one of the most commonly utilized methods for producing metal components [6]. LPBF provides substantial advantages in advanced manufacturing. It enables the fabrication of complicated geometries and designs based on digital CAD software with small and dense structures with high mechanical properties [7]. This makes it ideal for industries needing custom components, like the aerospace, medical, and automotive sectors [8,9]. However, it is difficult to fabricate large components due to equipment limitations; therefore, small components are produced and then joined using welding methods such as tungsten inert gas (TIG) and laser welding techniques.

Limited research has been conducted on the weldability of AMed components when using the TIG technique. Huysmans et al. [10] studied the optimization of TIG-welded parameters for 316L ASS fabricated by LPBF. They reported that the AMed 316L ASS components have good weldability at a current of 96 A, a welding speed of 80 mm/min, and a gas flow rate of 12 L/min. Furthermore, Mohyla et al. [11] studied the mechanical properties of TIG-welded joints for 316L ASS pipes fabricated by LPBF with variable welding parameters. It was observed that carbides which were originally precipitated in the base metal (BM) were dissolved. Therefore, the hardness was reduced by 30% in the fusion zone (FZ) compared to the BM. Khedr et al. [12] investigated the effect of build orientation on the mechanical properties and microstructure of TIG-welded joints in 316L ASS plates fabricated using the LPBF method. The study included different orientations: horizontal, inclined at 45°, and vertical. The results demonstrated that ultimate tensile strength increased as the build orientation shifted from 90° to 0°, while hardness resistance decreased. This variation in hardness is attributed to the higher cooling rates observed after the welding process. The kernel average misorientation (KAM) map results indicated that the vertically built specimens exhibited higher misorientation, which reflects a higher cooling rate following the welding process.

Laser welding offers advantages over TIG welding, including the use of the same heat source as the LPBF technique. This commonality allows laser welding to integrate seamlessly with LPBF, potentially reducing the overall manufacturing cycle time significantly. During laser welding, solidification occurs as epitaxial growth from the substrate. Therefore, grain orientation has great effects on the microstructure of FZ, and, consequentially, effects on the mechanical properties [13]. In addition, Feng et al. [14] investigated the influence of building orientations (BOs) (0°, 45°, and 90°) on the microstructure and mechanical properties of laser-welded joints in additively manufactured 300M steel. The study showed that as the BO shifted from 0° to 90°, the columnar-to-equiaxed-dendrites transition (CET) was increased in the FZ. In the 0–0° joint, dendrites grew in a columnar manner on both sides. In the 0–45° joint, full columnar growth occurred on the 0° side, with CET occurring at the weld center of the 45° side. The 0–90° joint displayed CET on both sides, forming an equiaxed dendrite zone at the weld center, which is attributed to higher cooling rates, as discussed in reference [15]. Casalino et al. [16] investigated the mechanical properties and microstructure of laser-welded joints in LPBF-fabricated 316L ASS. They found these joints exhibited a cellular and columnar dendritic microstructure with irregular grain orientations, showing no porosity but lower microhardness and tensile properties compared to the BM.

It is well known that LPBF process parameters, particularly build orientation, significantly impact the microstructure and mechanical properties of welded plates fabricated through additive manufacturing. This study examines the relationship between mechanical properties, such as hardness properties and the microstructural evolution of base metals built with different building orientations (0° and 90°). Additionally, the study investigates the indentation behavior of fusion zones in welded joints with 0–0° and 90–90° orientation combinations produced using different welding techniques, specifically TIG and laser welding.

## 2. Materials and Methods

### 2.1. LPBF Specimen Manufacture and Heat Treatment

The 316L ASS plates were manufactured using the LPBF technique. The raw material, in the form of a powder alloy, consisted of spherical particles with a size range of 10–45 μm. The chemical composition of the powder was measured using an ARL 9800 XP X-ray spectrometer (Thermo Fisher, Waltham, MA, USA) and is detailed in Table 1.

**Figure 1:**
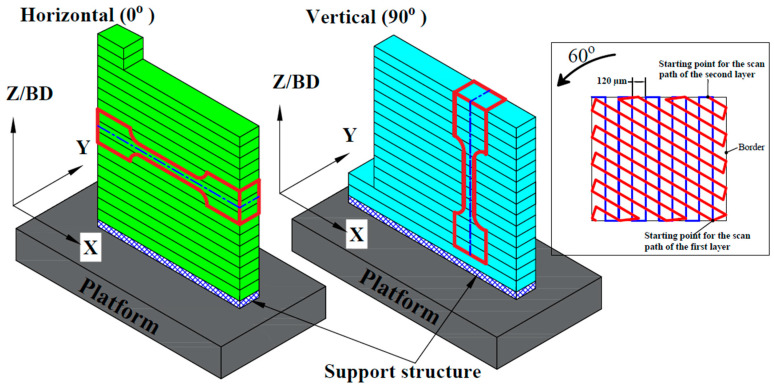
Schematic drawing of 3D specimens during manufacturing process with different building orientations. The laser track was rotated by 60° after each layer.

The specimens were fabricated using SLM 280 HL printing equipment (SLM Solutions, Lübeck, Germany), which employs a (Yb: YAG) laser system characterized by a 1030 nm wavelength and a power output (***P***) of 200 W. The scanning speed (***V***) was set at 800 mm/s. A zig-zag hatching pattern was used, with a 60° rotation angle between each layer, as illustrated in Figure 1. The distance between each scan path, referred to as the hatch space (***H***), was set at 120 µm, while the layer thickness (***T***) was set at 30 µm. Argon gas was supplied continuously at a pressure of 12 mbar and a flow rate of 7.5 m/s to avoid oxidation of the powder. The printing parameters were selected based on optimized parameters reported in the literature [12,14,17,18,19]. The volumetric energy density (**E_v_**) was approximately 70 J/mm^3^, as calculated according to Equation (1), as follows [20,21]:

(1) $$Ev=\frac{P}{V*T*H}$$

The specimens were printed with different BOs: a horizontal orientation (BO-0) and a vertical orientation (BO-90). Figure 1 illustrates the main difference between these orientations during the printing process, showing the cutting of tensile test specimens for each orientation. In the BO-0 specimens, the tensile force was applied at a 0-degree angle relative to the built layers. In contrast, in the BO-90 specimens, it was applied at a 90-degree angle to the built layers. The plates were 60 mm in length, 70 mm in width, and 2.5 mm in thickness. Subsequently, the plates were heat-treated in a Nabertherm N41/H chamber furnace at 1000 °C for one hour, followed by quenching in water [22,23].

### 2.2. Welding Processes

#### 2.2.1. TIG Welding Technique

**Figure 2:**
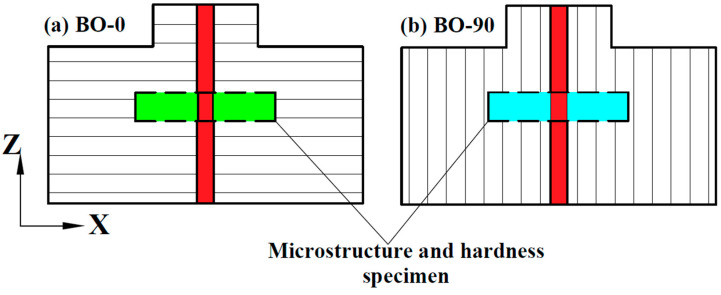
Schematic drawing of welded plates with a butt joint.

The specimens were welded in butt joint types with the TIG welding technique using ESAB TIG 4300I AC/DC equipment (Welding equipment, Esabvagen, Sweden). The welding process was performed in different directions to the built layer; i.e., the welding direction was perpendicular and parallel to the built layer for the BO-0 and BO-90 specimens, respectively, as shown in Figure 2. Pure argon gas was used as a shielding gas with a flow rate of 12 L/min. A filler metal of ER316L, with a diameter of 1.6 mm, was used to fill the 1 mm gap between two plates, and its chemical composition is presented in Table 2.

Table 3 shows the welding parameters and the corresponding heat input employed during welding as optimized in the literature [9,10]. The heat input was calculated according to Equation (2), as follows [24,25]:

(2) $$\mathrm{Heat}\;\mathrm{input}\;(\mathrm{kJ}/\mathrm{mm})=\;\frac{\eta *I*V}{S*1000}$$

where ***η*** is the efficiency of the welding joint, which is 60% in the TIG welding process according to the BS-EN1011-1 [26,27], ***I*** is the welding current (A), ***V*** is the voltage (Volts), and ***S*** is the travel speed (mm/s).

#### Delta Ferrite Measurement

The delta ferrite content of FZs was measured using a ferrite scope model (MF300FM, Cambridge, UK) following the standard procedure [28]. Additionally, a Schaeffler constitution diagram for the stainless-steel weld metal was utilized to predict the delta ferrite content according to the chemical composition throughout the FZ [29]. The chemical composition of the FZs was measured via the optical emission spectroscopy model (FOUNDRY-MASTER Pro, OXFORD INSTRUMENTS, High Wycombe, UK).

#### 2.2.2. Laser Welding Technique

Three-dimensional plates were welded employing a laser welding technique using a 6-axis industrial robot equipped with a Yb: YAG disc laser emitting system with a wavelength of 1030 nm. The welding direction was the same as that used for the TIG configuration, as illustrated in Figure 2. Argon was used as a shielding gas with a flow rate of 30 L/min. The settings and parameters of the welding process are presented in Table 4, according to optimized ones reported in the literature [16,20,30,31,32]. The energy input was calculated according to Equation (3) [33], as follows:

(3) $$\mathrm{Energy}\;\mathrm{input}=\frac{\;Laser\;power}{welding\;speed}$$

## 3. Mechanical Properties and Microstructure Evaluation

### 3.1. Microstructure Evaluation

Specimens for evaluating microstructure were prepared utilizing a CNC wire-cutting EDM machine, model FH-300C (Kunshan Ruijun Machinery Co., Ltd., Jiangsu, China). The direction of cutting was perpendicular to the welding line, as shown in Figure 2. After the cutting process, the specimens were embedded in epoxy with resin, and then prepared using mechanical grinding via silicon–carbide sandpapers of varying sizes (P240 to P2000 grain/cm^2^). Finally, the specimens were polished by alumina suspension. Additionally, electrochemical etching was performed using an electrolytic oxalic acid solution, following ASTM E407-07 [34] guidelines. The solution consisted of 10 g of oxalic acid dissolved in 100 mL of filtered water. The specimens were submerged in this etchant for 30 to 40 s, with an applied voltage of 6 V and a current of 1.5.

Optical micrographs were captured using an optical microscope (Olympus PMG 3, Waltham, MA, USA) and laser microscopy (LM), employing the KEYENCE laser confocal scanning microscope, VK-X200 (KEYENCE Corporation, Osaka, Japan).

### 3.2. Microhardness Measurements

Indentation hardness measurements were conducted using a micro-indentation hardness (H_IT_) tester (CSM instruments: Needham, MA, USA) with a diamond Berkovich indenter for different zones, including the base metal (BM), heat-affected zone (HAZ), and fusion zone (FZ). An accelerated load ranging from 0 to a maximum force of 2 N was maintained for 15 s.

## 4. Results and Discussion

### 4.1. Microstructure Characterization

#### 4.1.1. Microstructure of BM

**Figure 3:**
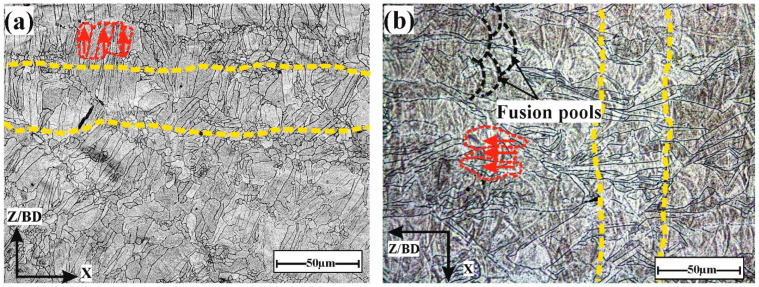
Microstructure of base metal with different building orientations: (**a**) BO-0 and (**b**) BO-90. The orientations of the columnar grains are highlighted with red dashed lines, and the layer boundaries are highlighted with yellow dashed lines.

In this section, we aim to provide a deeper understanding of how build orientation influences the microstructural characteristics of the BM, including grain size and orientation, and how these factors affect mechanical properties such as indentation resistance. Additionally, we examine the significant impact of build orientation on heat dissipation during both the printing and welding processes. Figure 3 illustrates the microstructure of BM with different Bos, as determined using a laser microscope. The microstructure showed two grain structures: columnar and equiaxed grains. This is in agreement with the literature [35]. Columnar grains (highlighted with red dashed lines) usually develop epitaxially from the substrate or previous layers, and then extend in the direction of the energy source/building direction. The columnar grains exhibited a perpendicular orientation on the *Z*-axis/layer boundary (highlighted with the yellow dashed line). The BO-0 sample showed elongated columnar grains that were aligned parallel to the building direction (Z/BD) and perpendicular to the *X*-axis. This alignment suggests a strong directional solidification influenced by the heat flow during the LPBF process. Additionally, this accelerated the cooling rate during the printing process, as described in the literature [36,37], which can impact mechanical properties such as tensile strength. The BO-90 sample demonstrated columnar grains that were aligned parallel to the Z/BD-axis and perpendicular to the *X*-axis. The strong vertical alignment of grains is characteristic of the upward build direction, which resulted in a pronounced columnar structure. This vertical orientation often leads to improved ductility owing to the tensile force in the direction of the grains. Equiaxed grains were formed due to a decreasing G/R ratio (where G represents the temperature gradient, and R denotes the solidification rate) from the fusion line to the upper section of the molten pool.

Figure 3b depicts the complex overlapping structure seen in the melt pools formed by multiple passages during the printing process (marked by black dashed lines). This overlap caused a more refined grain microstructure, which improved the material performance. Moreover, it assisted in filling gaps and cavities developed during earlier passes, thereby decreasing the material porosity/number of cavities and improving the density of the final products [38].

#### 4.1.2. Microstructure of TIG-Welded Joints

**Figure 4:**
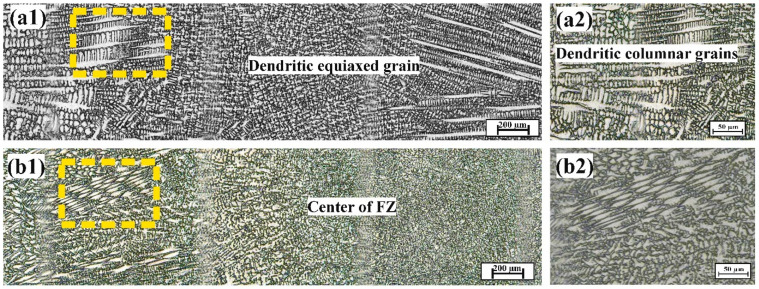
Microstructure images of the FZs produced by TIG welding for specimens with different welding directions, as follows: (**a**) BO-0 and (**b**) BO-90. (**1**) General views of FZs and (**2**) magnified views (highlighted with yellow dashed lines) of dendritic columnar grains.

Figure 4 presents laser microscopic images of FZs in specimens with different TIG welding directions relative to the built layer. Clearly, an epitaxial growth mechanism took place, where the grains of the BM acted as substrates for nucleation. Additionally, dendritic equiaxed grains formed in the middle of the FZs, with the grain size having been influenced by the cooling rate after welding at a constant heat input due to a heterogeneous nucleation mechanism, as reported in reference [39]. When the building orientation transitioned from 0° to 90°, the average width of the FZs decreased from 6.8 ± 0.5 mm to 4.6 ± 0.5 mm, respectively. Additionally, the average grain size of the equiaxed grains also decreased, measuring 8 ± 0.5 µm and 5 ± 0.5 µm for BO-0 and BO-90, respectively. This reduction in grain size indicates grain refinement and improved mechanical properties in accordance with the Hall–Petch relationship [40]. Notably, the width of the dendritic equiaxed region in the BO-90 specimen was larger than that of BO-0, resulting in enhanced mechanical properties, including increased indentation hardness.

**Figure 5:**
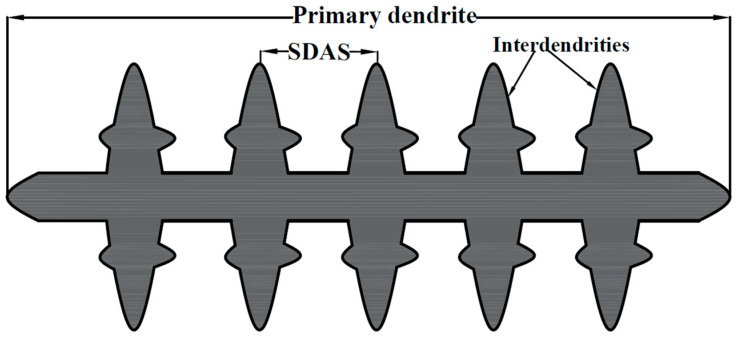
Schematic drawing identifies the welding dimensions, including dendrite length and secondary arm spacing.

Figure 5 illustrates the characteristics of dendrites formed in the FZ. At a lower cooling rate, grains had sufficient time to grow significantly, leading to increased dendrite length and interdendritic spacing. Consequently, this resulted in a reduction in indentation resistance, as reflected by the lower hardness values [41]. Olympus stream motion image processing software 2.5.2 was used to measure the dendrite length (primary length) and interdendritic spacing (secondary dendrite arm spacing, SDAS). The line extending from the center of the first dendrite to the center of n^th^ dendrite. This was determined using Equation (4), as follows [42,43]:

(4) $$SDAS=L_{Prim}*(n-1{)}^{-1}$$

Table 5 presents the results of measurements for FZ analysis. The results of the measurements revealed that the BO-0 welded joints exhibited a higher dendrite length and interdendritic spacing, measuring 178 µm and 10 µm, respectively, compared to the BO-90 joints, which measured 42 µm and 6 µm, respectively. This confirms that the building orientation significantly impacts the growth patterns of dendrites. In BO-0, the columnar dendrites grew more regularly, promoting a relatively slower cooling rate due to the uniform structure, allowing more efficient heat conduction away from the weld center. In contrast, in BO-90, the growth pattern became disordered, leading to an increased cooling rate, which aligns with the findings of Feng et al. [14].

#### Delta Ferrite Content

**Figure 6:**
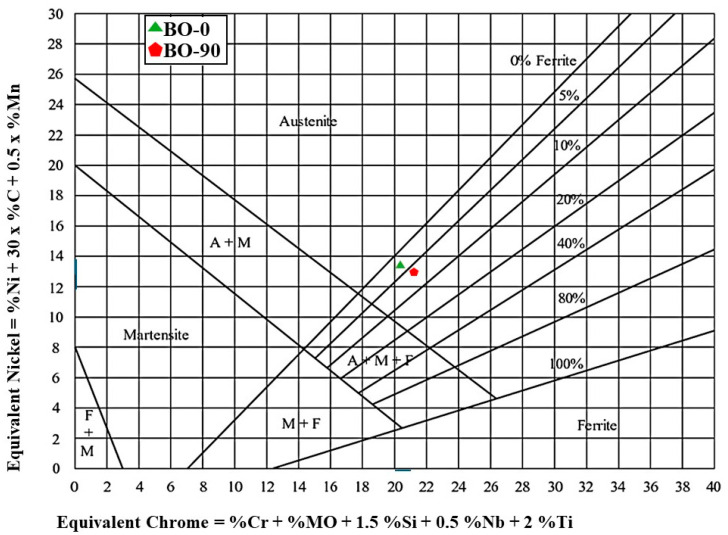
Schaeffler diagram showing the prediction of delta ferrite content in the FZs of TIG-welded joints.

Figure 6 illustrates the Schaeffler diagram used to predict the delta ferrite content in FZs, based on the chemical composition of the BM (presented in Table 1) and the welded joints (presented in Table 6). The BO-90 samples exhibited a higher delta ferrite content (6%) compared to the BO-0 samples (3%). Additionally, the ferrite scope measured volume fractions of delta ferrite of 1.9 ± 1.1and 4 ± 0.3% in the BO-0 and BO-90 joints, respectively.

The delta ferrite content in welded joints is influenced by the cooling rate after welding process. When the cooling rate is higher, as seen in the BO-90 sample, there is less time for the transformation of austenite into other phases, resulting in a higher retention of delta ferrite. Conversely, a lower cooling rate allows more time for austenite to transform into other phases, such as martensite or pearlite, thus reducing the delta ferrite content [44]. This may be the reason why the BO-0 joints, at lower cooling rates, showed a reduced delta ferrite content compared to the BO-90 joints.

#### 4.1.3. Laser-Welded Joints

**Figure 7:**
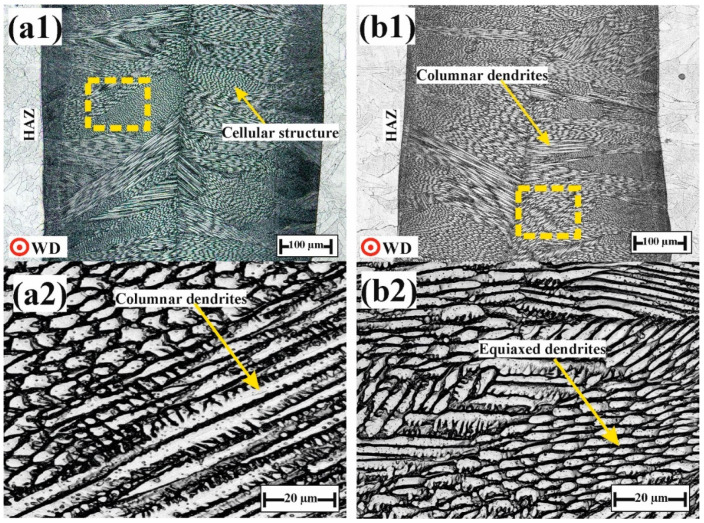
Laser microscope images for fusion zones produced by laser welding with different welding directions: (**a**) BO-0 and (**b**) BO-90. (**1**) General views of FZs and (**2**) magnified views (highlighted with yellow dashed lines).

Figure 7 illustrates the microstructure images obtained via LM for FZs produced by laser welding. The width of the FZs increased as the BO shifted from 90° to 0°, measuring 400 µm for BO-90 and 550 µm for BO-0. Clearly, the microstructure yielded a cellular structure and columnar dendrites. Additionally, within the columnar dendrites, both columnar and substructures grains were observed due to their preferred growth directions and differences in orientation. Throughout the dendritic structure, the columnar grains grew perpendicular to the fusion pool boundary, aligning with the direction of heat flow, which is in agreement with the literature [45]. Figure 7(a2,b2) illustrates high-magnification images of welded joints and show no defects produced during the welding process, such as porosity or solidification cracks. The center of the FZs was found to be primarily composed of equiaxed dendrite grains as a result of the low G/R ratio at the center of the FZ, where G represents the temperature gradient and R denotes the solidification rate. The average grain size of the equiaxed cellular structures in the FZs measured approximately 7 ± 3 µm in BO-0 and 5 ± 2 µm in BO-90. During laser welding, the energy input is significantly higher compared to the heat input during the printing process. This higher energy input results in a slower cooling rate after welding, providing more time for grain coarsening during solidification, which can potentially lead to reduced indentation hardness.

### 4.2. Effect of Building Orientation on Cooling Rate

#### 4.2.1. Effect of Building Orientation on Cooling Rate during Printing Process

**Figure 8:**
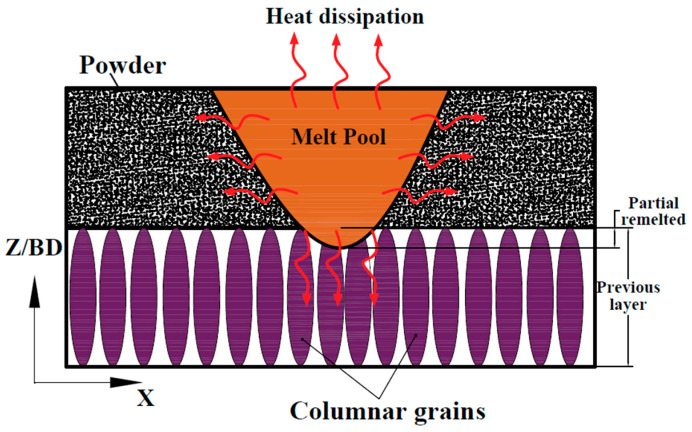
Heat dissipation during LPBF process.

Figure 8 illustrates the heat dissipation resulting from the melting of powder during the LPBF printing process, as described in the literature [36,37]. The columnar grains formed throughout the layer were aligned with the building direction and perpendicular to the fusion path/layer boundary in the direction of the source of energy, as discussed in the solidification of LPBF in [46,47]. During the printing process, the heat dissipation (highlighted by red arrows) from the fusion pool occurred in the direction of columnar grain initiation from the previous layer, which accelerated the heat dissipation and cooling rate. The higher cooling rate led to a smaller grain size, thereby enhancing strength and resistance to indentation (hardness properties). This is consistent with the microstructure of the BMs in Figure 3, where the grain size of BO-0 was smaller than that of BO-90.

#### 4.2.2. Effect of Building Orientation on Cooling Rate during Welding Process

During the welding process of the horizontal orientation specimen (BO-0), the welding direction was perpendicular to the built layer and parallel to the columnar grains. As a result, the columnar grains decelerated heat dissipation after welding. This deceleration reduced the cooling rate, leading to grain enlargement and increased the dendrite length and interdendritic spacing, which negatively impacted indentation resistance. In contrast, in the vertical orientation specimen (BO-90), the welding direction formed a 0-degree angle with the built layer and was perpendicular to the columnar grains. This accelerated the cooling rate after the welding process, leading to grain refinement and reduced interdendritic spacing. As a result, the indentation resistance was enhanced. This is consistent with the microstructure of the TIG- and laser-welded joints in Figure 4 and Figure 7, where the grain size of BO-90 was smaller than that of BO-0.

### 4.3. Hardness Results

#### 4.3.1. TIG-Welded Joints

**Figure 9:**
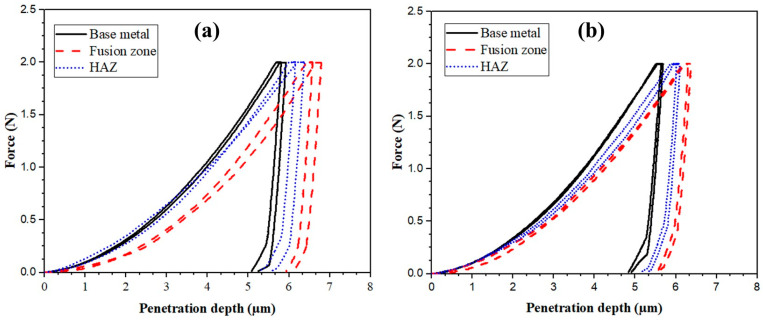
Load–penetration depth of micro-indentation hardness of TIG-welded joints: (**a**) BO-0 and (**b**) BO-90.

Figure 9 presents the micro-indentation hardness curves for TIG-welded joints. The measurements were taken across three zones: the BM, the heat-affected zone (HAZ), and the FZ. Table 7 presents the average values of H_IT_ and the corresponding penetration depths. Clearly, as the penetration depth (PD) increased, the material softened and the resistance to indentation decreased. The H_IT_ of the BMs measured 2.43 ± 0.17 and 2.45 ± 0.15 GPa in the BO-0 and BO-90 orientations, respectively. The hardness results of the BMs were consistent with those reported in the literature [36,37].

The PD of the FZs was greater than that of the BM, indicating that the indentation resistance of the FZs was lower than that of the BM. Specifically, the indentation resistance of the FZs was measured at 1.75 ± 0.22 GPa for BO-0 and 2.1 ± 0.09 GPa for BO-90. These values evidence the fact that during the welding process, the cooling rate of BO-90 was higher than that of BO-0. The higher cooling rate significantly affected the average grain size and dimensions of dendrites within the FZs, consequentially impacting the indentation hardness, as reported in the literature [48,49,50,51,52]. As mentioned in Table 5, the average grain size and interdendritic spacing for BO-90 were lower (5 ± 0.5 µm and 6 ± 1 µm, respectively) than those of BO-0 (8 ± 0.5 µm and 10 ± 1 µm, respectively). Therefore, the FZ of BO-90 exhibited a narrower penetration depth and higher hardness value.

**Figure 10:**
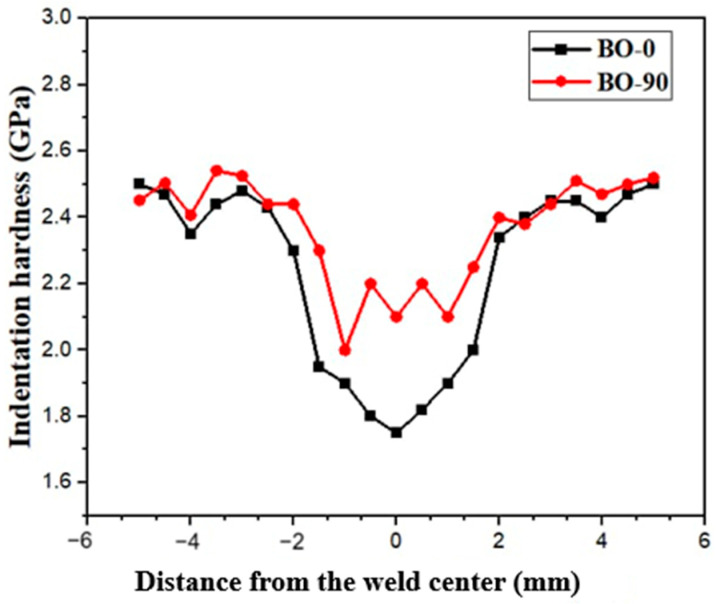
Profile of indentation hardness within the welded joints produced by TIG welding.

Figure 10 presents the microhardness profiles (H_IT_) for the BM, HAZ, and FZ in the TIG-welded joints with different BOs. The hardness profiles of the samples exhibit notable variations within the BM, HAZ, and FZ. The variations in hardness values are attributed to the different solidification rates during processing. For instance, the LPBF process led to a higher solidification rate compared to the TIG welding process [53,54]. This difference in solidification rates resulted in a finer microstructure in the LPBF products, while coarser grains were observed in the FZs after the TIG process. Furthermore, grain coarsening occurred in the HAZ, leading to reduced hardness in the HAZ compared to the BM structure. As a result, the hardness values of the BMs were higher than those observed in the HAZ and FZ.

#### 4.3.2. Laser-Welded Joints

**Figure 11:**
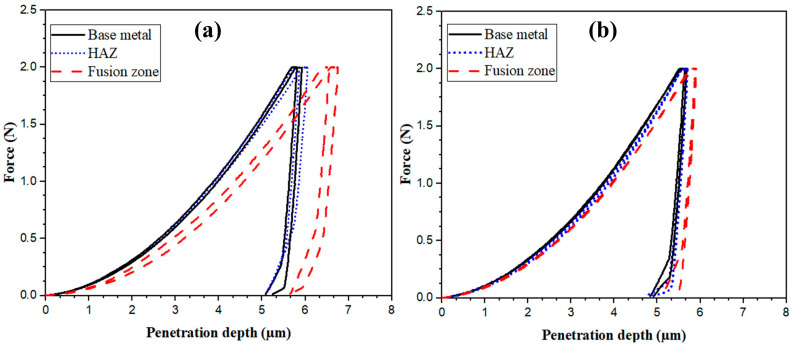
Load–penetration depth of micro-indentation hardness of laser-welded joints: (**a**) BO-0and (**b**) BO-90.

Figure 11 shows the micro-indentation hardness curves of the laser-welded joints. The H_IT_ values and the corresponding penetration depths are presented in Table 8. In the FZ, the indentation resistance of the laser-welded joints was higher than that of their counterparts, the TIG-welded joints. Notably, the indentation resistance in the FZ of the BO-90 specimens had the highest recorded value at 2.35 ± 0.11 GPa, surpassing that of the BO-0 specimens, which was 1.9 ± 0.16 GPa. This finding aligns with the greater cooling rates observed in BO-90 specimens and is consistent with the hardness results of the TIG-welded joints.

**Figure 12:**
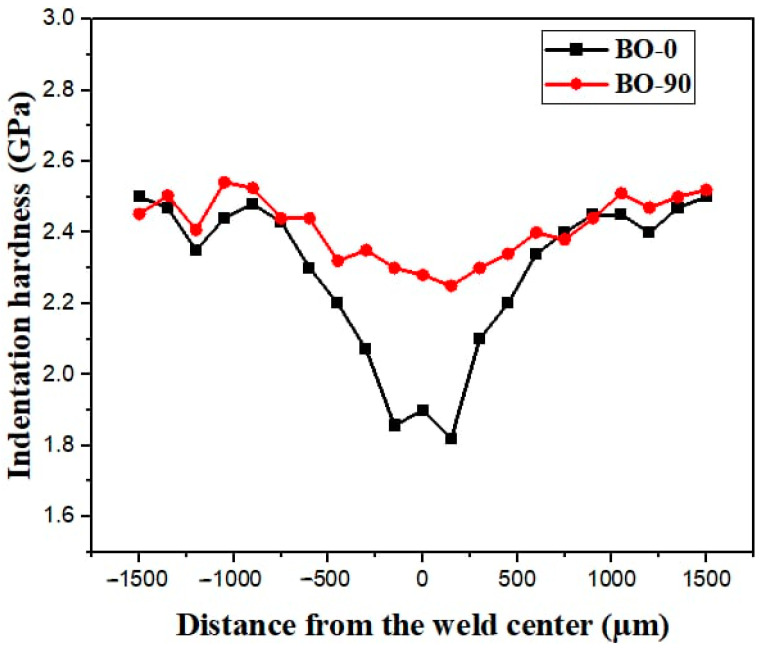
Profile of indentation hardness within welded joints produced by laser welding.

Figure 12 illustrates the micro-indentation hardness profiles of laser-welded joints across the BM, HAZ, and FZ. The FZs in laser-welded joints exhibited higher hardness than those in the TIG-welded joints, primarily due to the higher cooling rates associated with the laser welding process. Additionally, the laser beam used in laser welding employs a Yb: YAG disc laser emitting system, similar to the one used in printing BMs via LPBF. This resulted in the formation of a fine cellular structure in the FZ. Consequently, laser-welded joints demonstrated greater hardness in the FZ compared to TIG-welded joints, consistent with findings in the literature [55].

### 4.4. Summary of Results

**Figure 13:**
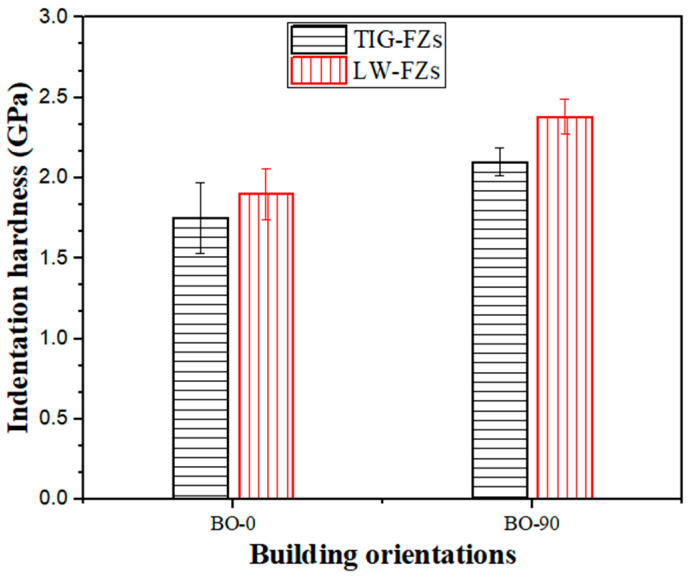
Indentation hardness values in FZs are produced by TIG and laser welding techniques.

Figure 13 illustrates H_IT_ hardness values in the FZs of TIG- and laser-welded joints. Clearly, the indentation hardness of FZs produced by TIG was lower than that of those produced by laser welding, despite the variation in the building direction of the printed BM. This increased hardness is attributed to the cellular structure and the fine grains produced by laser welding (as shown in Figure 7), which contrasts with the coarser dendritic structure resulting from TIG welding (as shown in Figure 4). The microstructural refinement in FZs achieved through laser welding significantly enhanced the mechanical properties of the additively manufactured 316L ASS welded joints.

Additionally, with the increase in the building orientation of the BM from 0 to 90°, the hardness values of the FZs were increased in both techniques. This is attributed to the higher cooling rates in BO-90 specimens, since the welding path is parallel to the printing direction of BO-90 specimens, resulting in greater heat dissipation after welding processing. Therefore, BO-90 displayed hardness values greater than those of BO-0 specimens.

## 5. Applications and Future Prospectives

Based on the current results, it is apparent that the building direction of additively manufactured 316L austenitic stainless-steel plates directly affects the microstructure and hardness characteristics of the welded joints, due to variations in the cooling rate after welding processing. It is recommended that the effect of different building orientations on the mechanical behavior of welded joints made of additively manufactured 316L austenitic stainless steel be considered. Although the TIG welding technique is commonly employed in many industrial applications, laser welding often results in narrow FZs with a negligible HAZ. The microstructure of the FZ in TIG-welded joints displayed the presence of a duplex structure consisting of delta ferrite and austenite, whereas the FZ of laser-welded joints depicted cellular structures with a single austenite phase. Thus, practitioners from the industry can decide the design and welding processing of components fabricated by additive manufacturing according to the selected application.

Regarding future prospectives, we recommend a comparison of the mechanical behavior of welded joints made of conventional (as-cast, rolled, etc.) and additively manufactured 316L austenitic stainless-steel plates with different building orientations. Therefore, a comprehensive study and comparison of the performance of traditionally and additively manufactured 316L stainless steels according to their mechanical and microstructural properties is encouraged to be carried out.

## 6. Conclusions

In the present study, 316L austenitic stainless-steel plates were additively manufactured using the LPBF technique with two orientations: horizontal (0°) and vertical (90°). The printed plates were joined using TIG and laser welding techniques, performed according to the direction of the built layer. The microstructure in the BM and FZ was examined using optical and laser microscopes, while the mechanical properties of the joints were assessed through hardness measurements via the micro-indentation hardness approach. The following conclusions were drawn:

- The building orientation significantly impacts the grain morphology of the base metal, affecting both grain size and orientation. For example, columnar grains tend to align with the build direction, forming perpendicularly to the fusion path.
- The dimensions of dendrite arms and grain size in the FZ of TIG-welded joints are influenced by the building orientation of the BMs. When the welding direction is parallel to the built layer, as observed in BO-90 specimens, the FZ shows finer interdendritic spacing and a smaller grain size compared to those welded perpendicularly to the built layers, as observed in BO-0. Specifically, the finest dendritic arm spacing (SDAS) was noticed in BO-90 welded joints, with an average value of 5 ± 0.5 µm, whereas this was recorded as 8 ± 0.5 µm in BO-0 joints.
- In both welding techniques, the indentation hardness values within the fusion zones varied depending on the welding direction relative to the build direction. BO-0 joints exhibited lower indentation hardness compared to BO-90 joints. This is likely attributed to the higher cooling rate experienced by BO-90 joints during the welding process compared to BO-0 joints.
- The higher cooling rate following the welding process results in finer structures within the fusion zone (FZ), directly influencing the indentation hardness values of the welded joints. Specifically, the FZ of the BO-90 orientation exhibited higher hardness, measuring 2.1 ± 0.09 GPa and 2.35 ± 0.11 GPa in TIG and laser welding, respectively. These values surpass the hardness of the FZ in BO-0 joints, which was recorded as 1.75 ± 0.18 GPa and 1.9 ± 0.16 GPa in TIG and laser welding, respectively.
- The similarity of laser beam characteristics between the printing process and laser welding technique offers the advantage of producing welded joints with a unified microstructure. This approach results in a finer microstructure, which in turn leads to increased indentation hardness in laser-welded joints compared to those produced by TIG welding.

## Figures and Tables

**Table 1:** Chemical composition of the powder used for printing AISI 316L, wt.%.

Element	Cr	Ni	Mo	Si	Mn	C	Nb	Ti	N	Fe
wt.%	17.70	12.90	2.50	0.70	0.6	0.02	0.005	0.01	0.09	Bal.

**Table 2:** Chemical composition of ER316L filler metal.

Element	Cr	Ni	Mo	Si	Mn	C	P	S	Cu	Fe
**wt.%**	18.0	12.50	2.60	0.40	1.51	0.01	0.02	0.01	0.1	Bal.

**Table 3:** Tungsten inert gas welding parameters and setting.

Current in Ampere (A)	Voltage (V)	Welding Length (mm)	Welding Time (s)	Travel Speed (mm/s)	Heat Input (kJ/mm)
100	12	60	30	2	0.36

**Table 4:** Laser welding process parameters and settings.

Laser Power (Watt)	Optical Diameter (µm)	Focal Point Level (mm)	Welding Speed (mm/s)	Energy Input (J/mm)
3000	200	−1	60	50

**Table 5:** Dimension analysis of fusion zones produced using a TIG technique.

Condition	Width of Fusion Zone (mm)	Width of Equiaxed Grain Region (mm)	Analysis of Fusion Zones		
			Average Grain Size (µm)	Average Dendrite Length (µm)	SDAS (µm)
BO-0	6.8 ± 0.5	1.1	8 ± 0.5	178 ± 4	10 ± 1
BO-90	4.6 ± 0.5	2.6	5 ± 0.5	42 ± 4	6 ± 1

**Table 6:** Chemical composition of fusion zones for specimens with different building orientations.

Condition	Cr	Ni	Mo	C	Mn	Nb	Ti	Si	Cr_eq_	Ni_eq_
**BO-0**	17.0	12.2	2.35	0.0143	1.32	0.088	0.0108	0.601	20.32	13.42
**BO-90**	17.8	11.8	2.33	0.0174	1.19	0.0927	0.0229	0.635	20.96	13.14

**Table 7:** Results of micro-indentation hardness tests and penetration depth (PD) for TIG-welded joints.

	BO-0			BO-90		
	BM	FZ	HAZ	BM	FZ	HAZ
**H_IT_ (GPa)**	2.43 ± 0.17	1.75 ± 0.22	1.95 ± 0.22	2.45 ± 0.15	2.1 ± 0.09	2.2 ± 0.19
**PD (µm)**	5.69 ± 0.2	6.7 ± 0.2	6 ± 0.2	5.61 ± 0.13	6.1 ± 0.15	5.82 ± 0.12

**Table 8:** Results of micro-indentation hardness tests and penetration depth (PD) for laser-welded joints.

	BO-0			BO-90		
	BM	FZ	HAZ	BM	FZ	HAZ
**H_IT_ (GPa)**	2.43 ± 0.15	1.9 ± 0.16	2.28 ± 0.08	2.45 ± 0.08	2.35 ± 0.11	2.42 ± 0.1
**PD (µm)**	5.75 ± 0.2	6.6 ± 0.16	5.85 ± 0.17	5.70 ± 0.13	5.88 ± 0.16	5.77 ± 0.17

## Data Availability

The original contributions presented in the study are included in the article, further inquiries can be directed to the corresponding authors.
